# Utilizing Artificial Intelligence in Critical Care: Adding A Handy Tool to Our Armamentarium

**DOI:** 10.7759/cureus.15531

**Published:** 2021-06-08

**Authors:** Munish Sharma, Pahnwat T Taweesedt, Salim Surani

**Affiliations:** 1 Internal Medicine, Corpus Christi Medical Center, Corpus Christi, USA; 2 Internal Medicine, University of North Texas, Dallas, USA

**Keywords:** artificial intelligence in medicine, critical care medicine, ai and machine learning, mechanical ventilation, sepsis

## Abstract

We have witnessed rapid advancement in technology over the last few decades. With the advent of artificial intelligence (AI), newer avenues have opened for researchers. AI has added an entirely new dimension to this technological boom. Researchers in medical science have been excited about the tantalizing prospect of utilizing AI for the benefit of patient care. Lately, we have come across studies trying to test and validate various models based on AI to improve patient care strategies in critical care medicine as well. Thus, in this review, we will attempt to succinctly review current literature discussing AI in critical care medicine and analyze its future utility based on prevailing evidence.

## Introduction and background

The world continues to witness advancements in technology at a rapid pace. Artificial intelligence (AI) has added another dimension in this quest to improvise the technological armory at our disposal. With the advent of AI, the field of medicine has also seen a palpable enthusiasm. There is still a lot of inquisitiveness about the burgeoning role of AI in medicine. In this article, we specifically attempt to review the existing evidence regarding the role of AI in critical care in a succinct manner.

## Review

**Figure 1:**
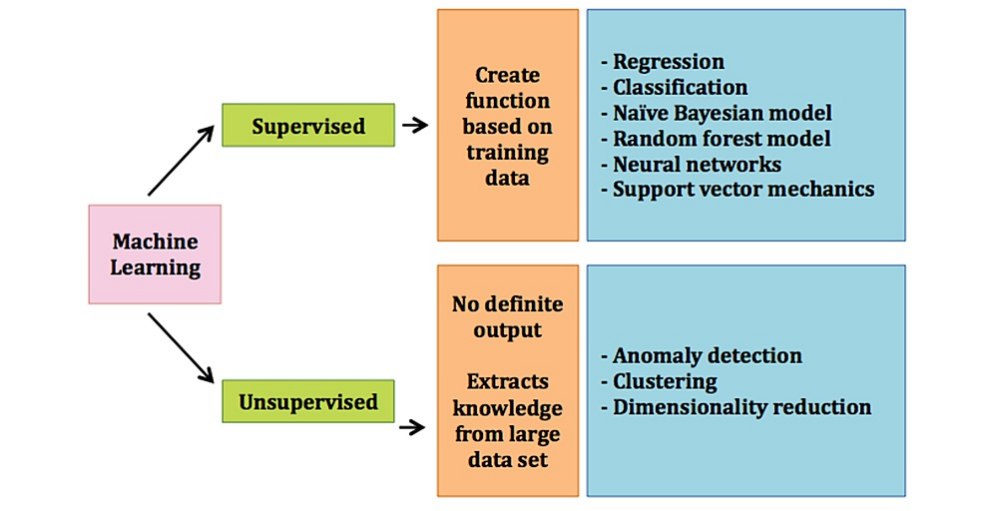
Machine learning and its subtypes

As per the definition found in Britannica by Copeland, AI is commonly referred to as a computer system with human intellectual features, e.g., reasoning, discovering, generalizing, and learning from prior exposure [1,2]. U.S Food and Drug Administration (US-FDA) has also stated in 2019 that AI has the potential to transform the healthcare industry by its ability to derive new information from the vast dataset that feeds into it [2,3]. Machine learning (ML) can be simply understood as a subset of an application of AI in which machines analyze and use a large dataset to produce unique algorithms capable of “statistical learning” as described by Gutierrez [2]. The use of ML has surged in critical care in the field of the discovery of drugs, diagnostic tools, medical imaging, and therapeutics amongst others. It can potentially help us better understand the vast set of data available to us in an intensive care unit (ICU) and apply it to tackle a multitude of medical conditions [2,4]. ML can be divided into two main models based on learning tasks, which are supervised and unsupervised learning algorithms. Supervised ML aims to create an algorithm that can predict output based on the specific nature of input provided to it. Supervised ML creates a function based on the training data and a specific set of training examples given to it that can accurately deliver results when exposed to unseen data [2,5]. Supervised ML includes regression, classification, naïve Bayesian model, random forest model, neural networks, and support vector mechanics [2]. Further discussion about these sub-classifications is beyond the scope of this review. Unsupervised ML involves extracting knowledge from a finite large data set that is unclassified and has no definite output. No specific instructions are provided to the algorithm regarding data processing. Anomaly detection, clustering, and dimensionality reduction are commonly used techniques of unsupervised ML (Figure 1) [2,6]. We do not intend to dwell on the details of these techniques in this review.

The use of computerized systems to aid in the management of critically ill patients is not an entirely new concept. The computer has been used in an ICU setting for various purposes, such as collecting patient’s history, monitoring patients’ vital signs, helping with the management of patients on mechanical ventilators, guiding physicians for the management of acute respiratory, and monitoring and interpreting blood gas values [7]. AI is fundamentally different from traditional computer algorithm in terms of how the rules or instructions are created. In traditional computing, a specific set of rules is created by the developer that intends to define an output based on each input provided. In sharp contrast to this, AI builds its own set of rules and creates an output based on previous numerous learning experiences [3]. The utilization of ML in an ICU setting is still in its infancy and a lot needs to be done before it can be amalgamated into our practice guidelines. More and more studies have been published to evaluate the ability of AI in the ICU setting. Many AI in critical care studies used datasets with a vast number of subjects to mainly predict the ICU mortality and duration of ICU stay. Moreover, AI has been studied to predict medical complications in ICU such as sepsis, acute renal failure, and Clostridium difficile infection. There have also been small studies using clinical data and ventilator signals to detect and facilitate mechanical ventilator-patient asynchrony problems [8-13].

Sepsis has certainly drawn a lot of interest from researchers over the past number of years. ML has been perceived as a potential tool to devise better management strategies in sepsis (Table 1).

**Table 1:** Studies highlighting the use of artificial intelligence in sepsis AISE - Artificial Intelligence Sepsis Expert; ANN - Artificial Neural Networks; ML - Machine Learning; n - Number

Author	Year	Study type (n)	Aim	Main Conclusion
Lukaszewski R.A. et al. [8]	2008	Retrospective (n=92)	Identify sepsis by ANN using cytokine and chemokine data	ANN was able to predict sepsis with high sensitivity and selectivity
Nemati S. et al. [9]	2018	Retrospective (n=27,527 in development vs n=42,411 in validation cohort)	Develop and validate ML algorithm for sepsis onset prediction	AISE algorithm precisely early predicted sepsis onset 4-12 hours prior to clinical recognition
Seymour C.W. et al. [10]	2019	Retrospective (n=63,858)	To develop and evaluate sepsis phenotypes	Four clinical phenotypes were detected relating to clinical outcomes

A study conducted by Lukaszewski et al. attempted to identify patients with sepsis even before they started developing symptoms [8]. This study involved 92 critically ill subjects who received interventions that posed a higher likelihood of sepsis for them. They used a panel of seven biomarkers measured in blood leukocytes mRNA by real-time reverse transcriptase-polymerase chain reaction technique. These biomarkers, including interleukin (IL)-1 beta, IL-6, IL-8, and IL-10, tumor necrosis factor-alpha, chemokine ligand (CCL)-2, and Fas-L were used as inputs. Neural network analysis was used to analyze the data and the results showed that ML can aid in using these seven biomarkers to predict sepsis with an accuracy of around 83.09% one to four days before the onset of clinical symptoms (sensitivity 91.43%). The predictive accuracy of the neural network was 94.55% based on comparative data obtained from 22 healthy volunteers [8]. In another study, researchers created an algorithm named Artificial Intelligence Sepsis Expert (AISE), i.e., sepsis onset prediction model. The study was a retrospective study including more than 31,000 patients admitted to the ICUs at Emory University hospital and 52,000 ICU patients from the Medical Information Mart for Intensive Care (MIMIC)-III ICU database for validation. Time series and static data from the electronic medical record were extracted. A total of 65 variables were assessed hourly and analyzed with the AISE algorithm at 12, eight, six, or four-hour window before the onset of sepsis. The significant contributing features to predict sepsis onset was demonstrated by the AISE algorithm. Using AISE, the area under the receiver-operating curve (AUROC) of the prediction model ranged from 0.83-0.85 for the 12, eight, six, and four-hour before sepsis onset. The AISE model showed high performance for real-time sepsis onset prediction in the critical care setting four to 12 hours preceding the usual clinical detection. The authors suggested that a prospective study was needed to evaluate the clinical implication of this sepsis onset prediction algorithm [9]. In another study, Seymour et al. sought to better define the differences among patients with sepsis. The study was performed using more than 60,000 patients with sepsis from three observational studies and three randomized controlled trials (RCTs). First, the derivation cohort was done, including 20,189 patients with sepsis. The validation cohort then included 43,086 patients. Subsequently, simulation models using data from RCT were performed. Four novel clinical phenotypes (alpha, beta, gamma, and delta) were derived from unsupervised learning. Using simulations, the benefits of treatment and harm were significantly different based on the phenotypic distributions. Each phenotype demonstrated dissimilar individual-specific variables, biomarker profiles, and clinical outcomes. The alpha phenotype, which included patients who received the least amount of a vasopressor, had the highest prevalence (n=6,625; 33%) among four phenotypes. Beta phenotype (n=5,512; 27%) contained relatively older patients, had more renal dysfunction and chronic comorbidities. Gamma phenotype had a higher number of patients with respiratory dysfunction and inflammation (n=5,385; 27%). Lastly, patients with delta phenotype (n=2667; 13%) were found to have more septic shock and hepatic function abnormalities [10].

Mechanical ventilation is another aspect of ICU that has been involved with ML in many studies (Table 2).

**Table 2:** Studies highlighting the use of artificial intelligence in mechanical ventilation ANN - Artificial Neural Networks; ML - Machine Learning; n - Number; PMV - Prolonged Mechanical Ventilation

Author	Year	Study type (n)	Aim	Main Conclusion
Parreco J. et al. [11]	2018	Retrospective (n=92)	Identify patients who will likely require PMV and tracheostomy	ML classifiers early detected patients with risk for PMV and tracheostomy
Hsieh M.H. et al. [12]	2018	Retrospective (n=3,602)	Create ANN to predict successful extubation in ICU	ANN effectively predicted successful extubation

A study by Parreco et al. was used to see if ML could be used for detecting individuals who have a higher risk for prolonged mechanical ventilation (PMV) and the need for tracheostomy. This study used the MIMIC-III database and defined PMV as a mechanical ventilator duration of longer than seven days. The gradient-boosting technique with a decision trees model was applied to generate an ML classifier. Six different severity of illness scores were calculated on the first day of admission to the ICU. Of 20,262 ICU stays with mechanical ventilation, 13.6% required PMV while 6.6% ended up getting tracheostomy. The ML classifier performed the prediction of PMV and tracheostomy with a mean ± SD AUROC of 0.820 ± 0.016, and 0.830 ± 0.011, respectively. For surgical ICU patients who required mechanical ventilation, ML classifiers predicted PMV and tracheostomy with a mean ± SD AUROC of 0.852 ± 0.017, and 0.869 ± 0.015, respectively. This study demonstrated the high specificity and accuracy of ML classifiers for both PMV and tracheostomy placement prediction [11]. Furthermore, Hsieh et al. conducted a study on over 3,000 subjects using a neural network to predict re-intubation with 72 hours of extubation. AUROC of 0.85 was obtained using this model. This study compared favorably against the AUROC of 0.54 for the existing gold standard parameter, i.e., rapid shallow breathing index [12].

ML has been used in a critical care setting to also assess the severity scores. Similar to Acute Physiology and Chronic Health Evaluation (APACHE) and the Simplified Acute Physiology Score (SAPS) is the existing assessment tool in a clinical setting as well as for research purposes. Their utility in clinical practice is still questionable [2,4,11]. Since the data used in these scoring systems are usually obtained from the first day of admission, events occurring during the subsequent days of admission may be overlooked and not reflect the dynamic nature of illnesses. The earlier and real-time assessment methods that can predict mortality risk would be valuable for physicians to help manage patients and prepare for goals of care discussion with family members. Awad et al. used the MIMIC-II database to predict in-hospital mortality for ICU patients. The researchers developed mortality prediction models for patients who were admitted to medical, surgical, and cardiac surgery ICU. Ensemble learning including Random Forest (RF), predictive decision trees, naive Bayes, and projective adaptive resonance theory models were conducted. In-hospital mortality was the primary outcome measured. Early Mortality Prediction for Intensive Care Unit-RF (EMPICU-RF) was shown to early predict mortality in patients who were admitted to the critical care unit. It demonstrated better performance than standard scoring systems, including Sequential Organ Failure Assessment (SOFA), SAPS-I, APACHE-II, National Early Warning Score (NEWS), and quick SOFA (qSOFA) based on the AUROC [13]. Cosgiff et al. utilized several ML techniques with sequential models to create an algorithm that can operate among every risk spectrum. Comparing to APACHE-IV, ML techniques obtained modest improvement in terms of AUROC for their sequential modeling. The authors concluded that these ML do enhance hospital mortality prediction [14]. Moreover, ML has been used in critical-ill patients for cardiac arrest prediction and outcome. Kim et al. demonstrated an ML model from 29,181 critically ill patients that early predicted cardiac arrest (AUROC of 0.886) and respiratory failure (AUROC of 0.869) six hours prior to the events which were higher than the modified early warning score (MEWS) and NEWS [15].

There have been studies utilizing ML in acute respiratory distress syndrome (ARDS) with some exciting outcomes (Table 3).

**Table 3:** Studies highlighting the use of artificial intelligence in acute respiratory distress syndrome (ARDS) ANN - Artificial Neuronal Network; ARDS - Acute Respiratory Distress Syndrome; CR - Clinical Recognition; EHR - Electronic Health Records; ML - Machine Learning, n - Number of Subjects, SAP - Severe Acute Pancreatitis

Author	Year	Study type (n)	Aim	Main Conclusion
You J.Y. et al. [16]	2020	Retrospective (n=1,305)	Compare rate and time of recognition of ARDS by ML with bedside CR	ML algorithm identified more cases of ARDS as compared to CR. No difference in the rate of identification
Le S. et al. [17]	2020	Retrospective (n=9,919)	Develop a model trained on patient data of health record to predict ARDS	Supervised ML can predict ARDS up to 48 hours before its actual onset
Sinha P. et al. [18]	2020	Retrospective (n=2,022 in training vs n=745 validation data)	Classify ARDS phenotypes by models trained on a clinical data set	ML models can accurately identify ARDS phenotypes
Zeiberg D. et al. [19]	2019	Retrospective (n=1,621 in training vs n=1,122 in test cohort)	Develop an ML approach to predict ARDS based on EHR	It is feasible to use the ML approach to risk-stratify patients for ARDS based on EHR
Fei Y. et al. [20]	2019	Retrospective(n=217)	Use ANN to predict and determine the severity of ARDS in SAP patients	Novel ANN can be used to predict ARDS in SAP

Since the delayed or missed diagnosis of ARDS can lead to unfavorable clinical outcomes, You et al. conducted a retrospective study to determine if ML can detect ARDS more frequently and earlier than clinicians [16]. They compared clinical recognition (CR) of ARDS with ML algorithm on 1,305 consecutive patients admitted from July 2017 to June 2018 in a hospital in New York City, USA. ML algorithm was able to recognize 73.5 % (959) cases of ARDS as compared to 33.2% (433) cases by CR. There was no statistically significant difference in ARDS recognition rate between the ML algorithm and CR [16]. In a retrospective analysis of 9,919 patients obtained from the MIMIC-III database, Le et al. tested gradient boosted tree models for early prediction of ARDS. The authors commonly utilized clinical variables and numerical data of radiology reports as the input to the model. The algorithm obtained AUROC values of 0.905 at onset and 0.827, 0.810, and 0.790 at 12, 24, and 48 hours before the ARDS onset [17]. Sinha et al. sighted the lack of phenotype identification with current point of care assays and aimed to develop models that could accurately classify ARDS phenotypes based on the readily available clinical data [18]. They used information from three randomized controlled trials as the training data while the fourth cohort served as the validation data. A gradient boosted ML algorithm was developed utilizing 24 different variables. The model accurately classified the ARDS phenotypes with AUROC of 0.95 with a 95% Confidence interval of 0.94-0.96 [18]. Zeiberg et al. aimed to develop an ML algorithm for the prediction of ARDS based on data extraction from electronic health records of the patients in a fully automated manner. Authors trained a model built for risk stratification of ARDS utilizing a cohort of 1,621 patients from a single center in the year 2016. Fifty-one out of the total cohort developed ARDS. They tested their model against a different cohort of 1,122 patients in 2017. Twenty-seven patients out of this cohort developed ARDS. A retrospective chart review was performed by critical care trained physicians for definite the diagnosis of ARDS. The model predicted ARDS with an AUROC of 0.81, 95% confidence interval of 0.73-0.88. It had a sensitivity of 56%, a specificity of 86%, and a positive predictive value of 9% [19]. In another study, Fei et al. attempted to use the artificial neuronal networks model (ANNs) to predict and determine the severity of ARDS in patients with severe acute pancreatitis (SAP) [20]. They created the ANNs model based on clinical data of 217 patients with SAP. They trained the ANNs model on 152 patients then validated it on 33 patients and tested it on 32 patients. ANNs model was found to have 87.5% sensitivity and 84.3% accuracy when subjected to the test set. ANNs model could identify ARDS in SAP with AUROC of 0.859 and there was a significant difference as compared to the logistic regression model [20].

Patients in an ICU are very prone to develop further complications during their course of treatment. AI has been studied in the prediction of complications and for risk stratification in patients in ICU [2]. There have been methods developed by Yoon et al. to predict instability during the ICU stay. The authors utilized logistic regression and random forest models from the variables of the electrocardiogram to predict instability with an accuracy of 0.81 and AUROC of 0.87 [21]. ML has been utilized to formulate risk stratification scores for entities like pulmonary embolism and acute kidney injury which are routinely encountered entities in an ICU [22,23].

Using ML in ICU has been shown to improve clinical outcomes by reducing the length of stay (LOS) and mortality (Table 4).

**Table 4:** Studies highlighting the use of artificial intelligence in the average length of stay and in-mortality prediction LOS - Length Of Stay; ML - Machine Learning

Author	Year	Study type (n)	Aim	Main results/conclusion
Shimabukuro et al. [24]	2017	Randomized controlled trial (n=92)	Develop severe sepsis prediction ML algorithm to reduce LOS and mortality rate	ML improved clinical outcome with the decrease in LOS and mortality by 20.6 and 12.4%, respectively
McCoy A. et al. [25]	2017	Prospective quality improvement (n=3,602)	Predict severe sepsis to compared sepsis-related LOS, mortality, and 30-day readmission	ML improved clinical outcome with the decrease in LOS, mortality, and 30-day readmission by 9.55, 60.24, 50.14%, respectively

Shimabukuro et al. conducted an RCT of adult medical-surgical ICU patients using ML to predict severe sepsis and outcomes [24]. One hundred and forty-two patients (75 in the control and 67 in the experimental group) participated in this study. ML algorithm was implemented to monitor, detect severe sepsis in the experimental group, and alert the care team to evaluate them. This study reported a significant reduction in the LOS (20.6%) in the experimental group (10.3 days) compared to the control group (13 days). Furthermore, in-hospital mortality decreased significantly in the experimental group (8.96%) compared to the control group (21.3%) [24]. McCoy and Das conducted a prospective quality improvement study to compare sepsis-related LOS, mortality, and 30-day readmission rate before and after the use of the ML algorithm [25]. ML algorithm was used to create a sepsis risk score to predict severe sepsis. The authors also evaluated the effectiveness of severe sepsis detection by ML using retrospective data from 1,665 encounters. ML (sepsis-3), ML (severe sepsis), systemic inflammatory response syndrome (SIRS), MEWS, qSOFA and SOFA demonstrated AUROC of 0.91, 0.96, 0.76, 0.55, 0.55, and 0.77, respectively [25]. Of 1,328 patients in ICU, progressive care unit, and the medical-surgical unit, 407 patients were in the pre-implementation period while 336, 381, and 204 patients were in the first, second, and steady-state post-implementation periods, respectively. With the use of ML-algorithm, in-hospital sepsis-related LOS, mortality, and 30-day readmission rate were reduced by 9.55%, 60.24%, and 50.14%, respectively [25].

Despite the promising results of the studies cited above, some challenges remain with AI. Since the results of these studies were mostly obtained and validated in a small patient cohort, it is difficult to predict if these results can be extrapolated to a larger population. The models studied need to be subjected to more rigorous well-designed prospective trials. Human expertise and proper technical resources need to be built to implement the ML into real-life clinical scenarios. Safe collection and use of public data in a digitalized format need to be ensured to garner trust surrounding the use of ML. Finally, uncertainties regarding the policy prescription and legislation to regulate ML in healthcare needs to be addressed as well. 

## Conclusions

It remains to be seen how the critical care fraternity is going to deal with the prospect of mingling with the AI in day-to-day patient care activities. Will there be a fear of going to be replaced or superseded by AI? Will it be rather perceived as another string to our bow? Looking at the prevailing pieces of evidence, AI promises to enhance efficacy and productivity in critical care. It can potentially better streamline our workflow and if deployed properly, increase our work pace and accuracy simultaneously. While AI hints at ushering a new era in healthcare, critical care physicians should still realize that we are solely responsible for making vital decisions for our patients. Advances in AI will not supplant the physicians but will likely complement our efforts in helping critically ill patients.

## References

[1] B.J. Copeland. Artificial intelligence (2021). Artificial intelligence. Encyclopædia Britannica. August 11.

[2] Gutierrez G (2020). Artificial intelligence in the intensive care unit. Crit Care.

[3] (2021). Machine learning (AI/ML)-based software (SAMD) as a medical device. https://www.fda.gov/media/145022/download.

[4] Mlodzinski E, Stone DJ, Celi LA (2020). Machine learning for pulmonary and critical care medicine: a narrative review. Pulm Ther.

[5] Mohri M, Rostamizadeh A, Talwalkar A (2018). Foundations of machine learning. https://cs.nyu.edu/~mohri/mlbook/.

[6] Hinton G, Sejnowski TJ (1999). Unsupervised learning: foundations of neural computation.

[7] Gardner RM, Scoville DP, West BJ, Bateman B, Cundick RM Jr, Clemmer TP (1977). Integrated computer systems for monitoring of the critically ill. Proc Annu Symp Comput Appl Med Care.

[8] Lukaszewski RA, Yates AM, Jackson MC (2008). Presymptomatic prediction of sepsis in intensive care unit patients. Clin Vaccine Immunol.

[9] Nemati S, Holder A, Razmi F, Stanley MD, Clifford GD, Buchman TG (2018). An interpretable machine learning model for accurate prediction of sepsis in the ICU. Crit Care Med.

[10] Seymour CW, Kennedy JN, Wang S (2019). Derivation, validation, and potential treatment implications of novel clinical phenotypes for sepsis. JAMA.

[11] Parreco J, Hidalgo A, Parks JJ, Kozol R, Rattan R (2018). Using artificial intelligence to predict prolonged mechanical ventilation and tracheostomy placement. J Surg Res.

[12] Hsieh MH, Hsieh MJ, Chen CM, Hsieh CC, Chao CM, Lai CC (2018). An artificial neural network model for predicting successful extubation in intensive care units. J Clin Med.

[13] Awad A, Bader-El-Den M, McNicholas J, Briggs J (2017). Early hospital mortality prediction of intensive care unit patients using an ensemble learning approach. Int J Med Inform.

[14] Cosgriff CV, Celi LA, Ko S (2019). Developing well-calibrated illness severity scores for decision support in the critically ill. NPJ Digital Medicine.

[15] Kim J, Chae M, Chang HJ, Kim YA, Park E (2019). Predicting cardiac arrest and respiratory failure using feasible artificial intelligence with simple trajectories of patient data. J Clin Med.

[16] You JY, Gong M, Chen JT (2020101016202008551). Machine learning algorithm increases recognition of ards. Critical care| volume 158, issue 4, supplement , A583, October 01.

[17] Le S, Pellegrini E, Green-Saxena A, Summers C, Hoffman J, Calvert J, Das R (2020). Supervised machine learning for the early prediction of acute respiratory distress syndrome (ARDS). J Crit Care.

[18] Sinha P, Churpek MM, Calfee CS (2020). Machine learning classifier models can identify acute respiratory distress syndrome phenotypes using readily available clinical data. Am J Respir Crit Care Med.

[19] Zeiberg D, Prahlad T, Nallamothu BK, Iwashyna TJ, Wiens J, Sjoding MW (2019). Machine learning for patient risk stratification for acute respiratory distress syndrome. PLoS One.

[20] Fei Y, Gao K, Li WQ (2019). Prediction and evaluation of the severity of acute respiratory distress syndrome following severe acute pancreatitis using an artificial neural network algorithm model. HPB (Oxford).

[21] Yoon JH, Mu L, Chen L, Dubrawski A, Hravnak M, Pinsky MR, Clermont G (2019). Predicting tachycardia as a surrogate for instability in the intensive care unit. J Clin Monit Comput.

[22] Banerjee I, Sofela M, Yang J (2019). Development and performance of the pulmonary embolism result forecast model (PERFORM) for computed tomography clinical decision support. JAMA Netw Open.

[23] Tran NK, Sen S, Palmieri TL, Lima K, Falwell S, Wajda J, Rashidi HH (2019). Artificial intelligence and machine learning for predicting acute kidney injury in severely burned patients: a proof of concept. Burns.

[24] Shimabukuro DW, Barton CW, Feldman MD, Mataraso SJ, Das R (2017). Effect of a machine learning-based severe sepsis prediction algorithm on patient survival and hospital length of stay: a randomised clinical trial. BMJ Open Respir Res.

[25] McCoy A, Das R (2017). Reducing patient mortality, length of stay and readmissions through machine learning-based sepsis prediction in the emergency department, intensive care unit and hospital floor units. BMJ Open Qual.

